# Immunodominant cytomegalovirus-specific CD8+ T-cell responses in sub-Saharan African populations

**DOI:** 10.1371/journal.pone.0189612

**Published:** 2017-12-12

**Authors:** Amna Malik, Emily Adland, Leana Laker, Henrik Kløverpris, Rabiah Fardoos, Julia Roider, Mai C. Severinsen, Fabian Chen, Lynn Riddell, Anne Edwards, Søren Buus, Pieter Jooste, Philippa C. Matthews, Philip J. R. Goulder

**Affiliations:** 1 Department of Paediatrics, University of Oxford, Oxford, United Kingdom; 2 Kimberley General Hospital, Kimberley, South Africa; 3 Africa Health Research Institute, AHRI, Durban, South Africa; 4 Laboratory of Experimental Immunology, Faculty of Health Sciences, University of Copenhagen, Copenhagen, Denmark; 5 University College London, Department of Infection and Immunity, London, United Kingdom; 6 Department of Sexual Health, Royal Berkshire Hospital, Reading, United Kingdom; 7 Department of Genitourinary Medicine, Northamptonshire Healthcare NHS Trust, Northampton General Hospital, Northampton, United Kingdom; 8 Oxford Department of Genitourinary Medicine, the Churchill Hospital, Oxford, United Kingdom; 9 Nuffield Department of Medicine, University of Oxford, Oxford, United Kingdom; Centre de recherche du CHUM, CANADA

## Abstract

More than 90% of children in Africa are infected with cytomegalovirus (CMV) by the age of 12 months. However, the high-frequency, immunodominant CD8+ T-cell responses that control CMV infection have not been well studied in African populations. We therefore sought to define the immunodominant CMV-specific CD8+ T-cell responses within sub-Saharan African study subjects. Among 257 subjects, we determined the CD8+ T-cell responses to overlapping peptides spanning three of the most immunogenic CMV proteins, pp65, IE-1 and IE-2, using IFN-γ ELISpot assays. A bioinformatics tool was used to predict optimal epitopes within overlapping peptides whose recognition was statistically associated with expression of particular HLA class I molecules. Using this approach, we identified 16 predicted novel CMV-specific epitopes within CMV-pp65, IE-1 and IE-2. The immunodominant pp65-specific, IE-1, IE-2 responses were all either previously well characterised or were confirmed using peptide-MHC tetramers. The novel epitopes identified included an IE-2-specific epitope restricted by HLA*B*44:03 that induced high-frequency CD8+ T-cell responses (mean 3.4% of CD8+ T-cells) in 95% of HLA-B*44:03-positive subjects tested, in one individual accounting for 18.8% of all CD8+ T-cells. These predicted novel CMV-specific CD8+ T-cell epitopes identified in an African cohort will facilitate future analyses of immune responses in African populations where CMV infection is almost universal during infancy.

## Introduction

Human cytomegalovirus (CMV) is a ubiquitous beta human herpesvirus. The prevalence of CMV infection is very high in developing countries [1] [2] and is endemic in sub-Saharan African populations with almost two-thirds of infants infected by 3 months of age and 85% infected by a year [3]. By adolescence CMV infection is virtually universal in sub-Saharan Africa [1].

CMV infection is usually asymptomatic but can lead to severe clinical complications in patients who are immunocompromised [4–6]. We focus here on the CMV-specific CD8+ T-cell response, since firstly, T-cell immunity plays an important role in controlling CMV infection [7] and preventing symptomatic, disseminated CMV disease [8–10] and secondly, the frequency of CMV-specific T-cell responses is unusually high, on average 10% of the CD4+ and CD8+ T-cell memory compartments in adult peripheral blood [11]. High magnitude CMV-specific T-cell responses have also been demonstrated in infancy [12].

Although the prevalence of CMV is high in sub-Saharan African populations from infancy, most previous studies defining CMV-specific CD8+ T-cell epitopes have focused on human leukocyte antigen (HLA) class I alleles that are common in white populations [13–18]. The aim of this study was therefore to identify the immunodominant CMV-specific CD8+ T cell epitopes in a study cohort of African subjects. In order to achieve this, we determined in >250 Southern African individuals the ex vivo responses by Interferon gamma (IFN-γ) enzyme-linked immunospot (ELISpot assay) to a panel of overlapping peptides spanning the pp65, 72-kDa immediate early (IE-1) and 86-kDa immediate early (IE-2) CMV proteins. These have previously been shown to be three of the most immunogenic CMV proteins [11] and are important for the structural assembly and replicative capacity of the virus [19].

We used a combination of ELISpot assays, peptide-HLA class I binding studies, bioinformatic prediction and peptide-HLA-Class I tetramer studies to predict and confirm novel CD8+ T cell epitopes in CMV. In this way, we have determined the immunodominant CMV-specific CD8+ T-cell responses in a Southern African cohort and defined 16 novel predicted CMV-specific CD8+ T-cell epitopes within pp65, IE-1 and IE-2, in three cases confirming the optimal epitope and HLA restriction using peptide-HLA class I tetramers.

## Material and methods

### Study subjects

We studied 257 HIV-infected ART-naive adult and paediatric subjects recruited via three previously described cohorts (Table 1):

1. Kimberley, South Africa, n = 69 [20]
2. Durban, South Africa, n = 84 [21]
3. Thames Valley cohort (southern African subjects attending outpatient HIV clinics in the Thames Valley area of the U.K, originating from Botswana, Malawi, South Africa, Kenya, Zambia and Zimbabwe), n = 104 [22].

**Table 1:** Cohort characteristics.

Cohort Name/ Location		Durban	TVC	Kimberley adults	Kimberley children	Total Adults	Total children
**Recruitment source**		Antenatal clinics	HIV clinics	Paediatric clinics (mothers of HIV-infected children)	Paediatric clinics	All cohorts	Kimberley
**HIV-status**		Positive	Positive	Positive	Positive	Positive	Positive
**No of individuals**		84	104	7	62	195	62
**HIV viral load (RNA copies/ml plasma)**	**Median**	5,450	3,848	34,706	22,600	4,300	22,600
	**IQR**	931–31,250	674–15,934	25,559–43,853	4,028–106,084	816–2,300	4,028–106,084
**CD4 T -cell count (cells/mm3)**	**Median**	581	510	528	1018	540	1,018
	**IQR**	415–699	362–615	461–622	724–1,381	396–659	724–1,381
**CD4 T-cell %**	**Median**	n/a	n/a	n/a	27%	n/a	27
	**IQR**	n/a	n/a	n/a	22%-30%	n/a	22–30%
**Age**	**Median**	26	n/a	n/a	6	n/a	6
	**IQR**	23–30	n/a	n/a	3–7	n/a	3–7

Ethics approval was given by the KwaZulu-Natal Review Board (Durban cohort); University of the Free State Ethics Committee, Bloemfontein (Kimberley Cohort) and the Oxford Research Ethics Committee (Durban, Kimberley and Thames Valley cohorts).

All adult subjects, or parents/guardians for children, provided written informed consent for participation.

In total, we used 62 paediatric (all from the Kimberley cohort) and 195 adult subject samples for ELISpot studies. We used an additional 37 subject samples from Durban cohort for NW8-tetramer staining. The samples were collected between 2009–2014. HIV viral load was measured using the Roche Amplicor version 1.5 assay according the manufacturer’s instructions; CD4+ T-cell counts were measured by flow cytometry. The CD4% is calculated as the percentage of live CD3+ CD4+ T-cells in total lymphocytes. The CD4% percentages in children were recorded at the recruitment site.

### HLA typing

DNA extraction was performed from whole blood using PureGene reagents (Qiagen, UK). Four-digit high resolution Sequence Based Typing of HLA-A, -B, and -C was performed from genomic DNA in the CLIA/ASHI accredited laboratory of William Hildebrand, PhD, (ABHI) at the University of Oklahoma Health Sciences Centre using a locus specific PCR amplification strategy and a heterozygous DNA sequencing methodology for exon 2 and 3 of the class I PCR amplicon. Relevant ambiguities [23] were resolved by homozygous sequencing.

We HLA-A/B/C typed 212 individuals, and in addition determined HLA-B-typing only on an additional 45 individuals.

### IFN-γ-ELISpot assay

*Ex vivo* CMV-specific CD8+ T-cell responses were screened in IFN-γ ELISpot assays using a panel of 401 overlapping 15-mer peptides (OLP), spanning the pp65, IE-1 and IE-2 region of CMV (OLP sequences available in S2 Table). IE-1 and IE-2 share part of their amino acid sequence; peptides 139–158 in IE-1 are identical to peptides 259–278 in IE-2, respectively.

Peptides were synthesised by Schafer-N (Denmark) in powder form at >80% purity. Following dilution to 400 μg/ml, peptide pools were produced with each pool containing 100 μl of 11–12 separate peptides, with each peptide present in two different pools. The peptide pools were generated as followed: 120 peptides in IE-1 making 22 pools; 143 peptides in IE-2 making 24 pools and 138 peptides in pp65 making 23 pools. A peptide was identified as reactive by positive responses in the two corresponding pools. OLPs identified by the megamatrix approach were then tested individually by ELISpot assay, depending on PBMC availability.

ELISpot assays were performed using sterile 96-well plates with a polyvinylidene fluoride membrane (MiIlipore, USA). The plate was pre-coated with anti-human IFN-γ monoclonal antibody (Mabtech, Sweden) in PBS at 1:2000 and incubated at 4°C overnight. Prior to use, the plate was washed 6 times with blocking buffer (1% heat-inactivated filtered foetal calf serum in PBS) and 50 μl of R10 (RPMI-1640, 100 μ/ml penicillin, 100μg/ml streptomycin, 2mM L-Glutamine, 10% heat-inactivated filtered foetal calf serum) was added to each well. Peptides were added to a final concentration of 40 μg/ml per well. Each plate contained two positive controls with 250 μg/ml of CMV lysate (Virusys) and four negative controls with no peptide/CMV lysate. Frozen PBMCs were thawed and 1x10^5^ were added per well. The plate was incubated for 14–16 hours in a humidified incubator at 37°C 5%CO_2_. After 6 washes with PBS, biotinylated anti-IFN-γ-antibody (Mabtech, Sweden) in PBS at 1:2000 was added and incubated at room temperature for 90 minutes in the dark. The plate was washed 6 times with PBS, incubated with streptavidin-alkaline phosphatase conjugate (Mabtech, Sweden) in PBS at 1:2000 for 45 minutes at room temperature in the dark. The plate was washed as before and developed using substrate colour solution (Bio-Rad laboratories, UK). Spots were counted using an automated ELISpot reader (AID ELISpot v4.0, Autoimmun Diagnostika, Germany). Background (>3 standard deviations above the mean of the 4 wells containing PBMC without a peptide) was subtracted from values of all wells, and the final result was expressed as the number of spot-forming cells (SFC) per 10^6^ PBMC. Positive responses were considered >100 SFC/10^6^ PBMC after background subtraction.

### Tetramer staining

Peptide-MHC tetramers were generated as previously described [24]. Cryopreserved PBMC (1 million per stain or less depending on availability) from the recipient were stained with PE-conjugated or APC-conjugated peptide-MHC tetramers, anti-CD3 Pacific Orange (Invitrogen, UK), anti-CD8 V450 (BD Biosciences, UK) antibodies and near-IR Live/Dead marker (Invitrogen, UK). Samples were analysed using an LSRII flow cytometer (BD, UK) collecting a minimum of 500,000 events and gating on singlets, lymphocytes, live cells and CD3+ CD8+ cells. Data were analysed using FlowJo version 10.0.7.

### Epitope prediction using *HLA-Restrictor*—A tool for prediction of HLA restriction elements and optimal epitopes within peptides

The *HLA-Restrictor* prediction tool [25] was employed to predict the epitopes within the OLPs with a significant HLA restriction. As input it requires four-digit HLA typing and the amino acid sequence of the overlapping 15mer peptide in question. It then predicts the peptide-MHC binding avidity for all possible 8-11mer peptides within the 15mer and on this basis, calculates the likelihood of the optimal epitope.

### Statistical analysis

Analysis was performed using Graph-Pad Prism v5.0c (GraphPad Software, Inc.).

Associations between peptide recognition and expression of particular HLA class I molecules were calculated with Fisher’s exact test using a 2x2 contingency table (p values shown in Tables 2 and 3). In order to correct for multiple comparisons, the Bonferroni correction was applied to give a p value threshold of 0.0009 (calculated as the uncorrected p value of 0.05 divided by the number of HLA class I alleles being studied, n = 55).

**Table 2:** HLA associations with the most targeted 15-mer OLP’s in CMV pp65, IE-1 and IE-2.

Protein	OLP	Sequence	HLA association	p value (Fisher’s)	Predicted optimal	Described/ Novel
pp65	66/67	QPFMRPHERNGFTVL	B*07:02	<0.0001	RPHERNGFTVL	Described^26^
		RPHERNGFTVLCPKN	B*42:01	<0.0001	RPHERNGFTVL	Novel
pp65	123/124	AGILARNLVPMVATV	A*02:01	<0.0001	NLVPMVATV	Described^26^
		ARNLVPMVATVQGQN				
IE-1	215/216	LSEFCRVLCCYVLEE	C*07:02	<0.0001	CRVLCCYVL	Described^27^
		CRVLCCYVLEELTSVM				
IE-2	342/343	LDNEKVRNIMKDKNT	A*30:01	<0.0001	KVRNIMKDK	Novel
		KVRNIMKDKNTPFCT				

The table shows the epitopes that were identified in our preliminary investigation using only the most immunogenic OLPs (OLP 66/67, OLP123/124, OLP215/216, OLP 342/343) in each protein. We identified two novel epitopes, for which we show FACS plots in Fig 2. Three epitopes have been previously described [26,27].

**Table 3:** HLA associations with the remaining 15-mer OLP’s in CMV pp65, IE-1 and IE-2 that were targeted by ≥4% of the study cohort.

Protein	OLP	Sequence	HLA association	p value (Fisher’s)	Predicted optimal	Described/ Novel
pp65	4	SVLGPISGHVLKAVF	B*81:01	<0.0001	GPISGHVL	Novel
pp65	4	SVLGPISGHVLKAVF	B*39:10	<0.0001	GPISGHVL	Novel
pp65	30	PLKMLNIPSINVHHY	B*35:01	<0.0001	IPSINVHHY	Described^29^
pp65	46/47	YYTSAFVFPTKDVAL	A*02:05	<0.0001	FVFPTKDV	Novel
pp65		AFVFPTKDVALRHW				
pp65	52	VCSMENTRATKMQVI	C*06:02	<0.0001	TRATKMQVI	Described^30^
pp65	53/54	ENTRATKMQVIGDQY	A*30:02	<0.0001	KMQVIGDQY	Novel
pp65		ATKMQVIGDQYVKVY				
pp65	104/105	TERKTPRVTGGGAMA	B*07:02	<0.0001	TPRVTGGGAM	Described^31^
pp65		TPRVTGGGAMAGAST				
pp65	131	YRIFAELEGVWQPAA	B*45:01	0.0008	AELEGVWQPA	Novel
IE-1	163	RHRIKEHMLKKYTQT	A*30:01	0.001	RIKEHMLKKY	Novel
IE-1	186	LQAKARAKKDELRRK	A*74:01	<0.0001	LQAKARAKK	Novel
IE-1	187	ARAKKDELRRKMMYM	B*08:01	<0.0001	ELRRKMMYM	Described^32^
IE-1	187/188	ARAKKDELRRKMMYM	B*18:01	<0.0001	DELRRKMMY	Novel
IE-1		KDELRRKMMYMCYRN				
IE-1	200	EIMAYAQKIFKILDE	A*23:01	0.0004	AYAQKIFKI	Novel
IE-1	212/213	ACMMTMYGGISLLSE	B*42:01	<0.0001	TMYGGISLL	Novel
IE-1		TMYGGISLLSEFCRV				
IE-1	226	KVFAQYILGADPLRV	C*07:02	<0.0001	QYILGADPL	Novel
IE-1	233	ESDEEEAIVAYTLAT	B*35:01	0.0007	EAIVAYTL	Novel
IE-1	234	EEAIVAYTLATAGVS	B*40:02	0.001	EEAIVAYTL	Novel
IE-2	374/375	TADACNEGVKAAWSL	B*44:03	<0.0001	NEGVKAAW	Novel
		CNEGVKAAWSLKELH				

The table shows additional predicted epitopes identified using the remaining 15mer peptides that were targeted by ≥4% of the study cohort. We identified an additional 14 novel predicted epitopes, and four previously published [29–32] epitopes.

## Results

### Immunodominant CMV-specific CD8+ T-cell responses within pp65, IE-1 and IE-2

**Fig 1:**
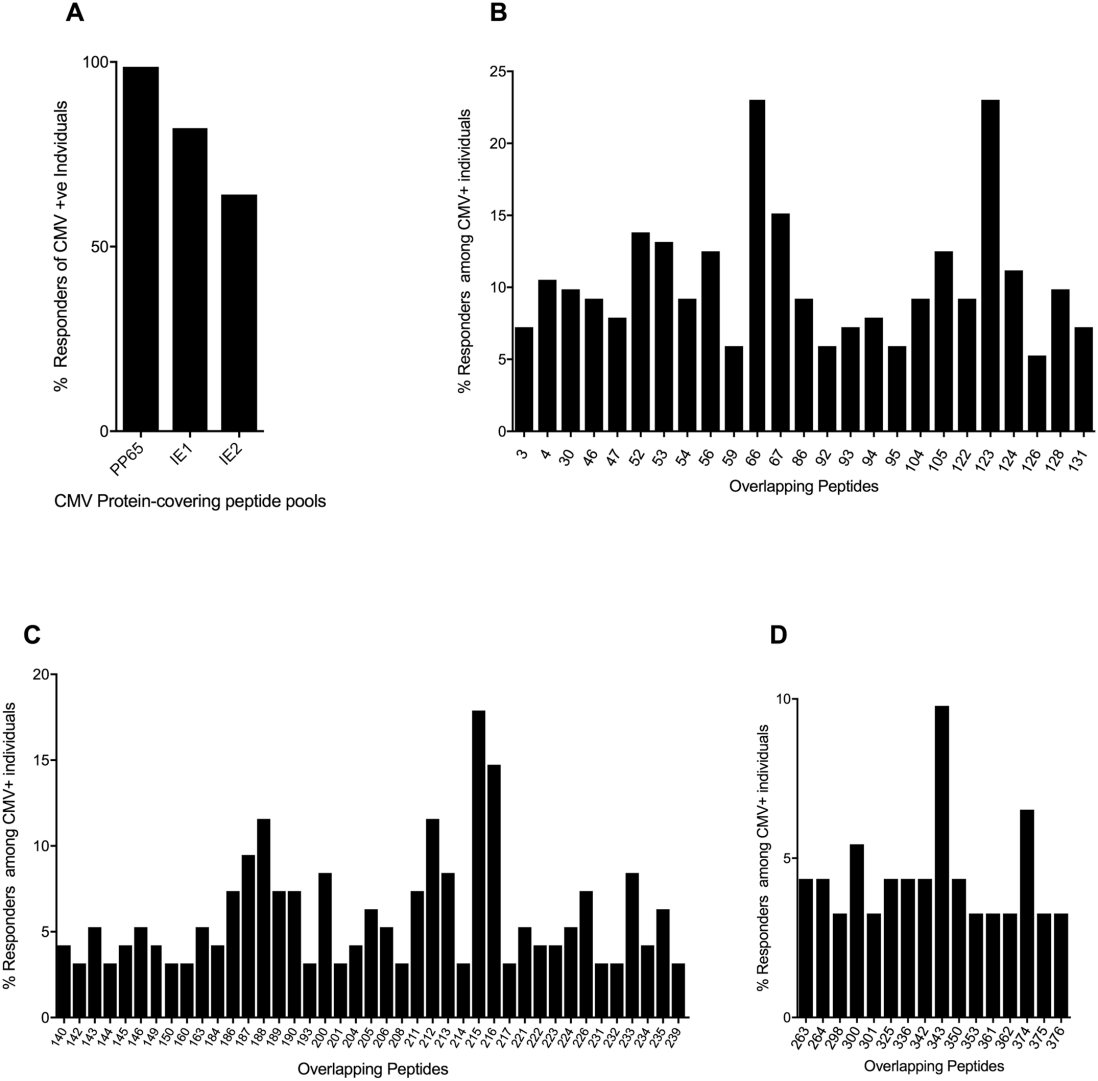
Immunodominant CMV-specific CD8+ T-cell responses within pp65, IE-1 and IE-2 measured by IFN-g ELISpot assay for CMV+ subjects. (A) Percentage responders to each protein-covering peptide pool. (B) Percentage responders among 152 CMV+ individuals to pp65 15mer overlapping peptides that were targeted by >4% of the study population. (C) Percentage responders among 95 CMV+ individuals to IE-1 15mer overlapping peptides that were targeted by >3% of the study population. (D) Percentage responders among 92 CMV+ individuals to IE-2 15mer overlapping peptides that were targeted by >3% of the study population.

All but two of the 257 HIV-infected subjects studied made responses to one or more of the three CMV proteins tested. The proportion of the individuals studied who showed responses to each of the three proteins tested was 98% for pp65 (n = 152), 82% for IE-1 (n = 95) and 64% for IE-2 (n = 92) (Fig 1A). Fig 1B–1D show the overlapping peptides that were targeted by more than 4% of individuals tested for pp65 and more than 3% of individuals tested for IE-1 and IE-2. Within each protein, certain 15mer overlapping peptides were immunodominant: within pp65, two peptides were recognised by 23% of the study population (pp65 OLP-66, and OLP-123), within IE-1, one 15mer was recognised by 17% of the study cohort (IE-2 OLP-215), and within IE-2, one 15mer was recognised by 10% of the subjects studied (IE-1 OLP-343) (Fig 1B–1D). The OLPs adjacent to immunodominant ones were also recognised, and the majority of the subjects making a response to these were within the group who recognised immunodominant OLPs. For example, 70% of the individuals responding to OLP-67 also responded to OLP-66, 88% of the individuals responding to OLP-124 also responded to OLP-123, 92% of the individuals responding to OLP-216 also responded to OLP-215 and 50% of the individuals responding to OLP-342 also responded to OLP-343. Recognition of two adjacent OLPs indicates the location of the 8-11mer optimal epitope, at least in part, within both 15mers.

**Fig 2:**
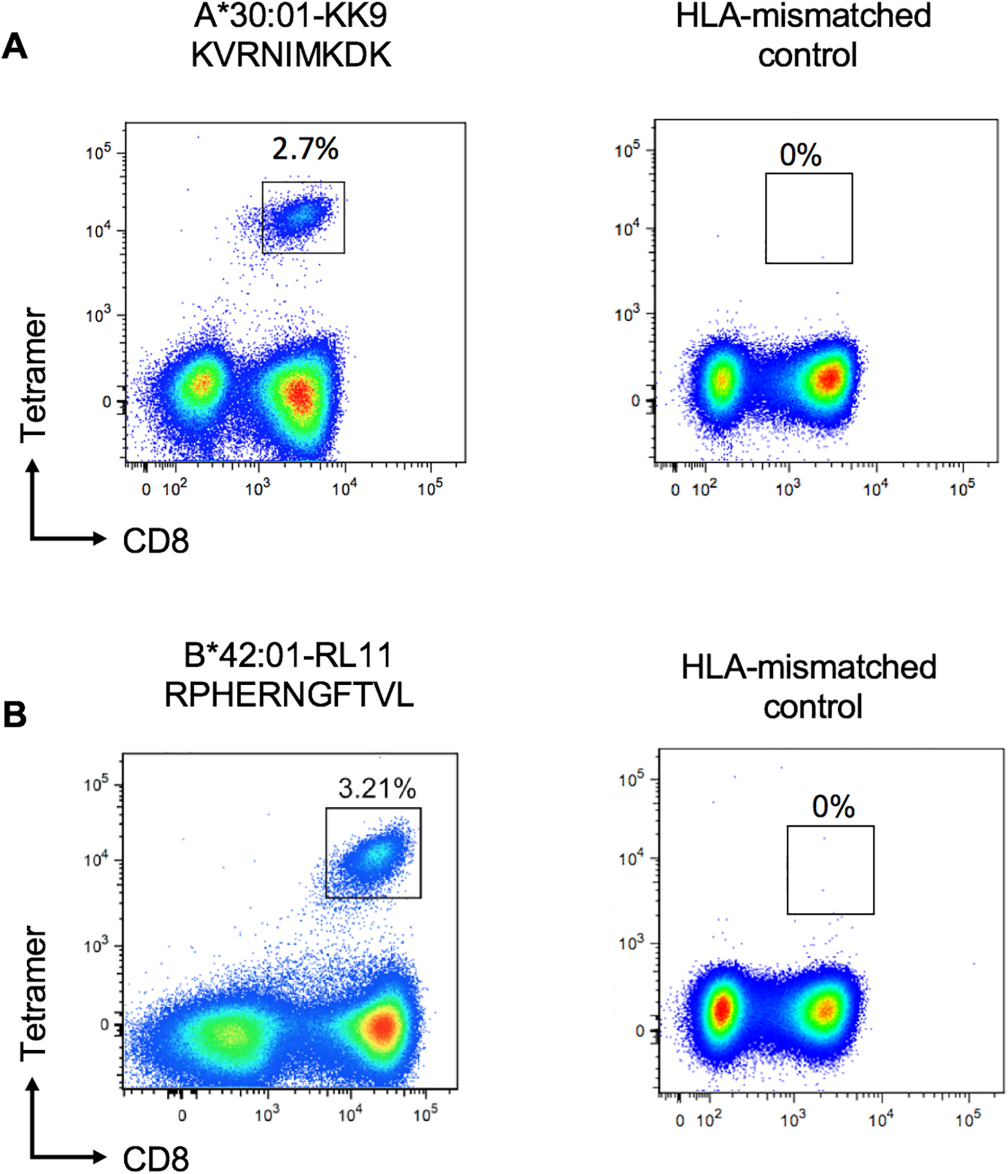
Validation of novel CD8+ T-cell epitopes in CMV using HLA-Class I tetramers. (A) Flow cytometry plot of CD8+ T-cell responses from a Durban cohort subject SK-251 (HLA-A*02:05, -A*30:01, -B*42:01, -B*58:01, -C*07:01, -C*017:01) to HLA-A*30:01-restricted IE-2-342/343 epitope-KK9 and an HLA-mismatched tetramer stain as a control. (B) Flow cytometry plot from a Durban cohort subject SK-331 (HLA-A*30:01, -A*66:01, -B*42:01, -B*58:02, -C*06:02, -C*017:01) to B*42:01-restricted pp65-66/67 epitope RL11 and an HLA-mismatched tetramer stain as a control. The plots show gated live CD3+ T-cells; the number shown above each gate is the percentage of live CD3+ CD8+ cells that are tetramer-specific.

Recognition of these 15mers in the ELISpot assays was in each case strongly associated with expression of particular HLA class I molecules (Table 2). An epitope predictor tool *HLA-Restrictor* [25] was then used to predict the optimal epitope from within the 15mers that would be presented by the relevant HLA class I molecule. This yielded 3 well-characterised and previously described epitopes [26,27] including the HLA-A*02:01-restricted epitope NLVPMVATV [26] and the HLA-B*07:02-restricted epitope RPHERNGFTVL[26], both in pp65. Two novel epitope candidates emerged also from this analysis, an HLA-B*42:01-restricted epitope RPHERNGFTVL in pp65 and an HLA-A*30:01-restricted epitope KVRNIMKDK within IE-2. The respective peptide-MHC tetramers were synthesised and used to stain PBMC in subjects who had shown responses to the 15mer peptides; in each case, these tetramers confirmed the optimal epitope and the HLA restriction of the response (Fig 2). One subject per epitope was tested with the corresponding tetramer.

This approach to defining the optimal CMV-specific epitopes within a Southern African study cohort therefore was validated for the 15mer overlapping peptides that were best recognised within the three CMV proteins studied.

### Identification of additional novel CD8+ T-cell epitopes within pp65, IE-1 and IE-2

Due to the encouraging results, we decided to additionally analyse the remaining 15mer peptides to which responses were observed in ≥4% of the study cohort. This threshold was chosen because, at <4% recognition, the number of subjects studied did not provide sufficient statistical power to detect associations between peptide recognition and expression of particular HLA class I molecules that would remain significant (p<0.05) after correction for multiple tests (uncorrected p value <0.0009: see Methods). In addition to the five epitopes described above (Table 2), we identified responses to 18 predicted CMV-specific CD8+ T-cell epitopes that were significantly associated with expression of particular HLA class I molecules (uncorrected p<0.0009, corrected p<0.05) (Table 3). Using epitope predictor tool, we found that 14 predicted epitopes are novel and four have been previously described in the literature (Table 3). In summary, across both experiments, we found that seven of the yielded epitopes had been previously described and the remaining 16 predicted epitopes are novel. As might be expected, the previously described epitopes are presented by HLA class I molecules that are highly prevalent in white populations whilst the novel predicted epitopes are restricted by HLA class I molecules that are in many cases broadly African-specific. The PF of the HLA alleles presenting the CMV-specific epitopes identified in this study are shown in S1 Fig (the figure shows the PF of HLA alleles presenting the CMV-specific epitopes in a representative white population [28] [www.allelefrequencies.net] and our African cohort).

### Closely related HLA I molecules shape distinct CMV-specific CD8+ T-cell hierarchies

**Fig 3:**
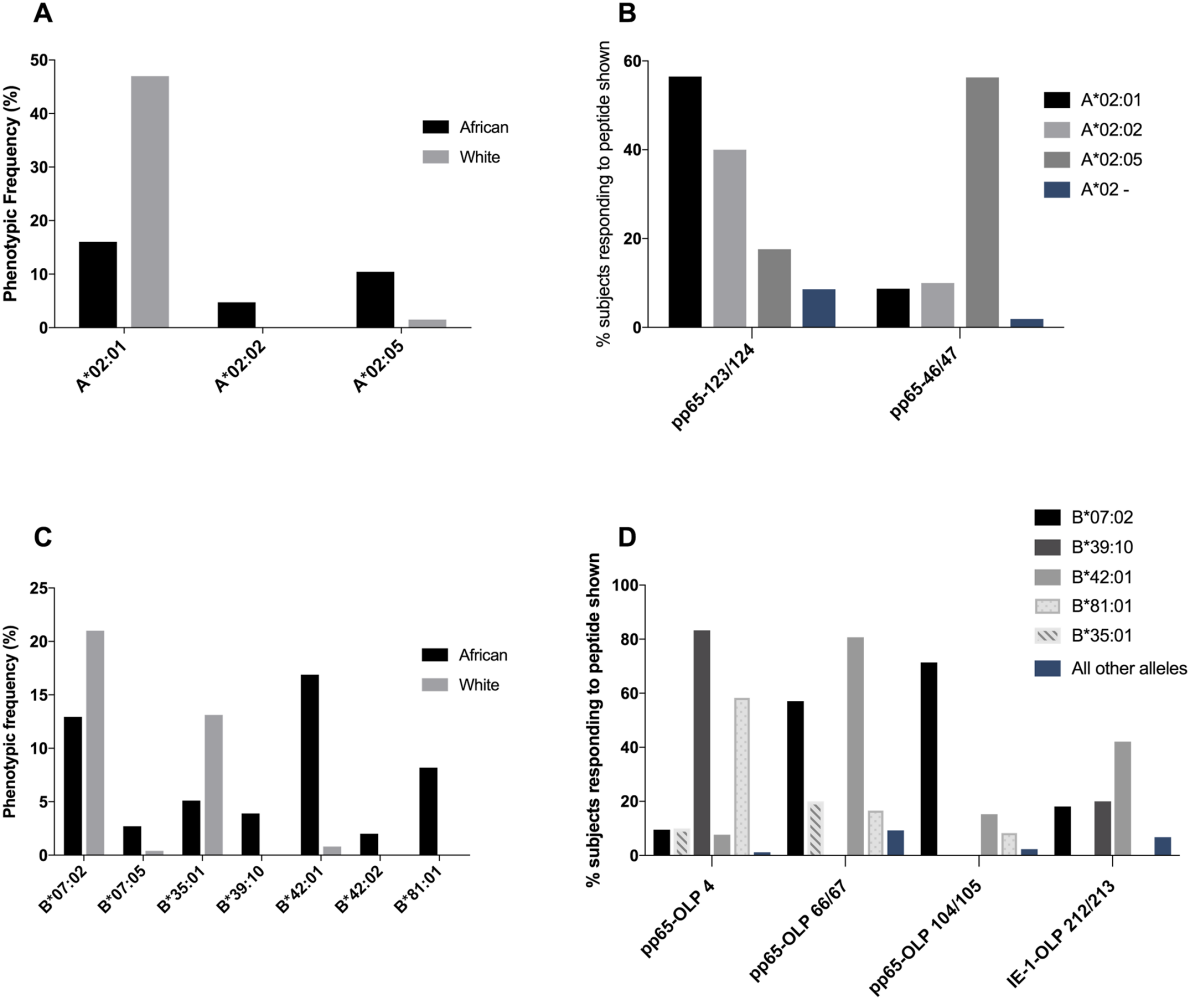
Phenotypic frequencies of closely related HLA I molecules and their impact on CMV specific CD8+ T-cell hierarchies. (A) The phenotypic frequency of some of the HLA class I molecules in A2 superfamily in white populations [28] and our African cohort. (B) The percentage of subjects with HLA alleles A*02:01 (n = 23), A*02:02 (n = 10) and A*02:05 (n = 16), responding to epitope (NLVPMVATV) in pp65-123/124 peptide and predicted epitope (FVFPTKDV) in pp65-46/47 peptide. (C) The phenotypic frequency of the HLA class I molecules in B07 superfamily in white populations [28] and our African cohort. (D) The percentage of subjects with B*07:02 (n = 21), B*35:01 (n = 10), B*39:10 (n = 6), B*42:01 (n = 26) and B*81:01(n = 12) alleles in our African cohort responding to predicted epitopes in pp65, GPISGHVL (OLP-4), RPHERNGFTVL (OLP-66/67) and TPRVTGGGAM (OLP-104/105). The percentage of subjects in our African cohort responding to predicted epitope in IE-1, TMYGGISLL (OLP-212/213) with HLA-B*07:02 (n = 11), B*35:01 (n = 6) B*39:10 (n = 5), B*42:01 (n = 19) and B*81:01(n = 1) alleles.

Comparison of the HLA class I molecules prevalent in white and sub-Saharan African populations in many cases reveals small differences in phenotypic frequency between the closely-related HLA alleles. Among HLA-A*02-positive white individuals, for example, 97% express HLA-A*02:01[28], whereas approximately 50% of HLA-A*02-positive individuals in our African cohort express HLA-A*02:02 or HLA-A*02:05, HLA-A*02 molecules that differ by 3 and 4 amino acids, respectively, from HLA-A*02:01(Fig 3A). However, it is clear from our study that the immunodominant HLA-A*02:01-restricted response is towards the pp65 OLP-123/124 peptide (NLVPMVATV), as described previously [26], whereas these data suggest that the immunodominant HLA-A*02:05-restricted response, more prevalent in Africans, is towards the pp65 OLP-46/47 peptide (FVFPTKDV) (Fig 3B).

Similarly, among HLA class I molecules within the B7 superfamily, only HLA-B*07:02-restricted responses have been well characterised to date. The HLA-B*07:02 allele is the most common allele in B7 superfamily [28] in white populations. There are two pp65-specific HLA-B*07:02-retricted epitopes that are both targeted by >50% of subjects expressing HLA-B*07:02. However, one of these two epitopes is clearly immunodominant among HLA-B*42:01-restricted CMV-specific responses, and neither is significantly targeted by subjects expressing either HLA-B*39:10, HLA-B*81:01 or HLAB*35:01 (Fig 3D). HLA-B*39:10, HLA-B*81:01 and HLA-B*35:01 alleles are part of the B7 superfamily and common African alleles. Thus, as might be expected and shown previously in relation to other virus-specific CD8+ T-cell responses [33] [34], the difference of even a few amino acids between closely-related HLA class I molecules can have a substantial impact on the CD8+ T-cell immunodominance hierarchy observed.

### Definition of a high frequency HLA-B*44:03-restricted IE-2-specific response

**Fig 4:**
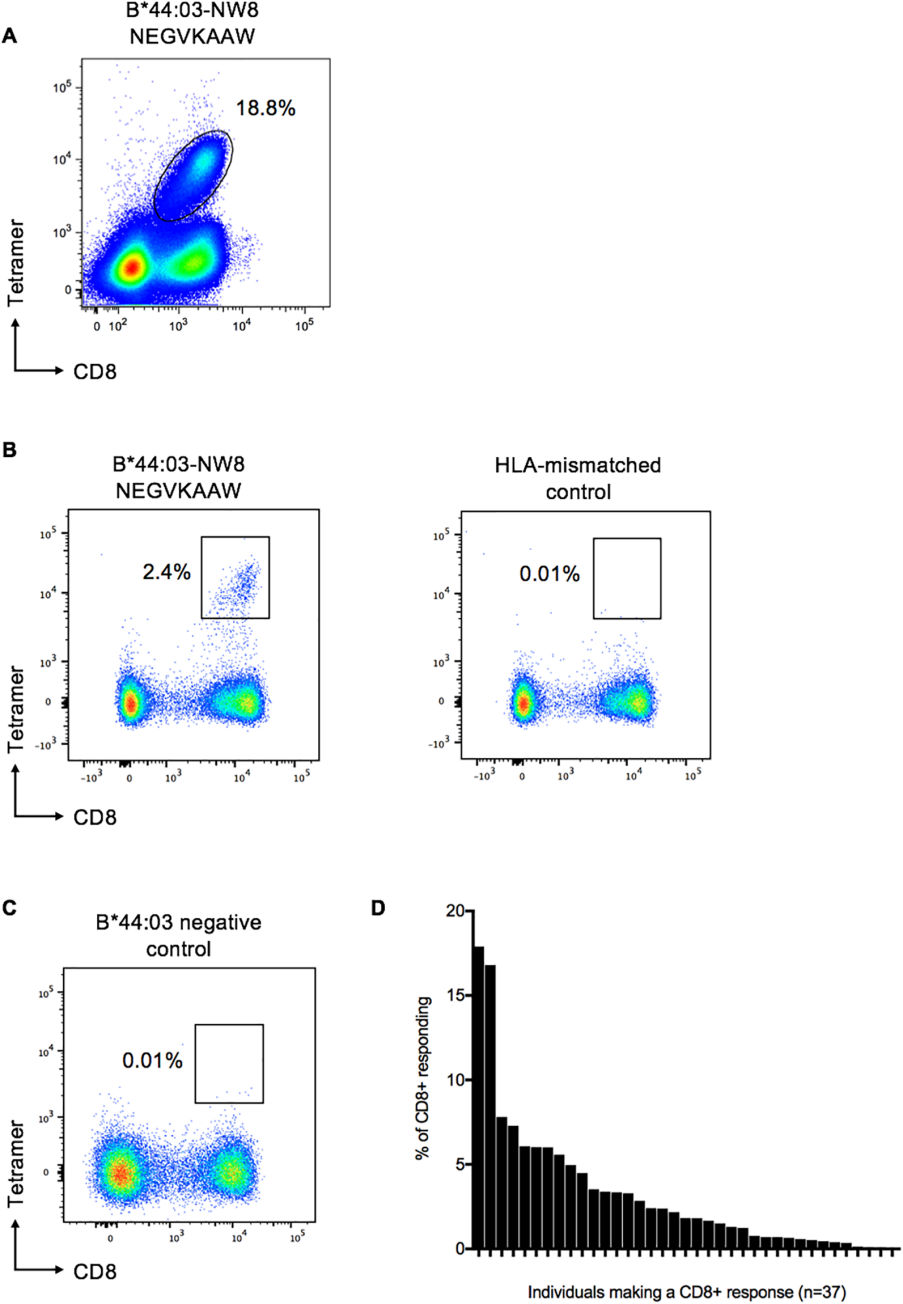
Individuals making CD8+ T cell responses against CMV IE-2 epitope HLA-B*44:03-NW8. (A) Representative FACS plot of a Durban cohort subject 202-30-0064 (expressing HLA-B*44:03/B*58:01) to HLA-B*44:03-restricted NW8 tetramer. (B) FACS plot of subject R048 (HLA-A*29:02, -A*6802, -B*15:03, -B*44:03, -C*02:10, -C07:01) with NW8 tetramer and HLA-mismatched tetramer. (C) FACS plot of a TVC subject R112 (HLA-A*23:01, -A*3002, -B*18:01, -B*18:01, -C*02:02, -C07:04) to HLA-B*44:03-restricted NW8 tetramer, as B*44:03-negative control. The FACS plots show tetramer-specific cells expressed as the percentage of live CD3+ CD8+ T-cells. (D) Percentage of live CD3+ CD8+ tetramer-specific T-cells in individuals expressing HLA-B*44:03 measured using the B*44:03-NW8 tetramer.

One of the novel epitopes identified in these studies is the epitope NEGVKAAW (NW8) in IE-2 restricted by HLA-B*44:03. The responses to this epitope were unusually high, even for CMV-specific CD8+ T-cell responses that are generally high magnitude responses, in one individual accounting for 18.8% of CD8+ T-cells (Fig 4A). As controls, we show representative FACS plots of a HLA-B*44:03 expressing individual stained with an HLA-mismatched tetramer and a non-HLA-B*44:03 expressing individual stained with the NW8-tetramer (4B-C). We tested a subset of 37 individuals from the Durban cohort with the NW8-epitope tetramer, that were not part of the ELISpot studies. Among the 37 subjects tested, 35 (95%) made responses above the positive threshold of >0.02% of CD8+ T-cells to this epitope, with a mean CD8+ T-cell response of 3.4% (Fig 4D).

## Discussion

The aim of this study was to define the immunodominant CMV-specific CD8+ T-cell responses in a study cohort comprising Southern African individuals, focusing on three of the most immunogenic CMV proteins pp65, IE-1 and IE-2. We validated some of these immunodominant responses using peptide-MHC-class I tetramers. Using this approach, we have identified 16 novel CMV-specific epitopes within CMV pp65, IE-1 and IE-2. Using a population of HIV-infected subjects, all of whom have sub-Saharan African origin, it has been possible, for the first time, to investigate the HLA restrictions for alleles most prevalent in sub-Saharan Africa. The resulting epitopes will underpin further understanding of CMV specific T-cell immunity, which is of particular interest considering the high frequency of CD8+ T-cells that recognize these epitopes.

One of the epitopes validated was an IE-2 epitope restricted by the predominantly African allele, HLA*B*44:03, that induced consistently high CD8+ T cell responses in individuals expressing HLA-B*44:03, in one individual approaching 19% of their CD8+ cells. Typically, during viral infections, the establishment of a memory T-cell population in response to an antigen is thought to involve expansion followed by contraction into a stable pool of memory cells, after the infection is cleared [35]. However, in CMV infection, CD8+ T-cells specific for certain epitopes do not contract but instead are maintained at high frequencies. This increase in CMV specific CD8+ T cell response has been termed ‘memory inflation’ [36]. ‘Memory inflation’ is a term derived from studies of murine cytomegalovirus (MCMV) that detected unusually high numbers of MCMV-specific CD8+ T-cells and eventual stabilization of memory CD8+ T-cells at high frequencies, in immune competent mice [36]. The underlying mechanisms of memory T cell inflation in humans are not yet fully elucidated. However, one feature described is restricted TCR usage [26][37]. We looked for evidence of memory inflation in our cohort but found no association between the size of CD8+ T-cell responses in relation to the age of the individuals. This may be due to the adults in our study cohort being relatively young (median age of 26 years).

HLA-B*44:03, a common allele in African populations, forms a part of HLA-B*44 supertype family. The predominant HLA-B*44 allele in white populations is HLA-B*44:02. Although these two HLA-B44 allotypes differ only at one position, residue 156 (HLA-B*44:02: 156-Asp; HLA-B*44:03: 156-Leu), the impact of this micropolymorphism appears to be that HLA-B*44:03 presents virtually all the peptides that are presented by HLA-B*44:02, plus some additional peptides not presented by HLA-B*44:02 [38]. The one HLA-B*44:02-positive subject tested made no IFN-gamma ELISpot response to the overlapping peptides containing NW8.

These studies were undertaken in a cohort of HIV-infected African subjects, prompting the question of whether the CMV-specific CD8+ T-cell responses might be affected. It is well established that, at extremely low absolute CD4 count (<50 CD4 T-cells/mm^3^) HIV-infected individuals are at risk of opportunistic CMV infections such as CMV retinitis [19]. However, the HIV-infected individuals studied did not include such severely immunocompromised subjects (the mean absolute CD4 count among the adults studied was 540 T-cells/mm^3^). Indeed, HIV-infected subjects who are infected with CMV typically have a higher magnitude CMV response than their HIV-uninfected counterparts [39]. For these reasons, we believe the identification of CMV-specific CD8+ T-cell epitope would not be substantially affected in such a study cohort. Further evidence for the validity of the overall approach comes from the identification of the previously well-characterised HLA-A*02:01-restricted NLVPMVATV and the HLA-B*07:02-restricted RPHERNGFTVL epitopes [26], as described above.

Although the work presented here involves a comprehensive study of peptide immunodominance against 3 CMV-encoded proteins in a large African cohort, there were some limitations beyond our control. We were not able to test all subjects for all three CMV proteins due to a lack of PBMC availability. Testing each protein for all subjects could help us identify more novel epitopes in pp65, IE-1 and IE-2. The ELISpot assays were performed without CD8+ T-cell enrichment so CD4+ T-cell cross reactivity with the overlapping peptides cannot be ruled out, however, the tetramer staining for some of the epitopes is unequivocal evidence for CD8+ T-cell specificity. The HLA class I associations with recognition of particular peptides is further evidence against these being CD4 T helper responses. A CMV negative control to test the tetramers would have been desirable but it is not feasible to find a CMV negative subject in an African cohort.

These new findings facilitate further analysis of CMV-specific CD8+ T-cell responses in African populations where most infants are CMV-infected within the first few months of life and approaching 90% are CMV-infected by 1 year of age. CMV is a major cause for concern in sub-Saharan African populations, due to high prevalence of HIV infection in these populations. The immunodeficiency caused by chronic HIV infection influences the consequences of co-infection, increasing the risk of long-term morbidity and mortality. Identification of novel epitopes reported here that are restricted by common HLA alleles could pave the way to immune-mediated prevention and/or therapy of CMV infection.

## Supporting information

S1 Fig Phenotypic frequencies of the class I HLA molecules in our African study cohort and a representative white population. The figure showing the phenotypic frequencies of HLA alleles highlighted in Tables 2 and 3. (A) Phenotypic frequencies of HLA-A molecules, (B) phenotypic frequencies of HLA-B molecules and (C) phenotypic frequencies of HLA-C molecules in our African cohort and a representative white population [28].
(TIFF)

S1 Table Phenotypic frequencies of all the class I HLA molecules in our African study cohort. (TIFF)

S2 Table Overlapping peptide sequences. (A) Sequences of overlapping peptides in pp65, (B) sequences of overlapping peptides in IE-1 and (C), sequences of overlapping peptides in IE-2.
(PDF)

## References

[1] Cannon MJ, Schmid DS, Hyde TB. Review of cytomegalovirus seroprevalence and demographic characteristics associated with infection. Reviews in Medical Virology. 2010;20(4):202–13. doi: 10.1002/rmv.655 20564615 10.1002/rmv.655

[2] Adland E, Klenerman P, Goulder P, Matthews PC. Ongoing burden of disease and mortality from HIV/CMV coinfection in Africa in the antiretroviral therapy era. Frontiers in Microbiology. 2015;6(1016). 10.3389/fmicb.2015.01016 PMC4585099 26441939

[3] Miles DJC, Sande MVD, Jeffries D, Kaye S, Ojuola O, Sanneh M, et al Maintenance of large subpopulations of differentiated CD8 T-cells two years after cytomegalovirus infection in Gambian infants. PLoS ONE. 2008;3(8). 10.1371/journal.pone.0002905 PMC2483415 18682836

[4] Deayton JR, Sabin CA, Johnson MA, Emery VC, Wilson P, Griffiths PD. Importance of cytomegalovirus viraemia in risk of disease progression and death in HIV-infected patients receiving highly active antiretroviral therapy. The Lancet. 2004;363(9427):2116–21. 10.1016/S0140-6736(04)16500-8 15220032

[5] Detels R, Leach C, Hennessey K, Liu Z, Visscher B, Cherry J, et al Persistent cytomegalovirus infection of semen increases risk of AIDS. The Journal of Infectious Diseases. 1994;169(4):766–8. 8133089 10.1093/infdis/169.4.766

[6] Griffiths PD. CMV as a cofactor enhancing progression of AIDS. Journal of Clinical Virology. 2006;35(4):489–92. doi: 10.1016/j.jcv.2005.10.016 16413825 10.1016/j.jcv.2005.10.016

[7] Moss P, Khan N. CD8+ T-cell immunity to cytomegalovirus. Human Immunology. 2004;65(5):456–64. doi: 10.1016/j.humimm.2004.02.014 15172445 10.1016/j.humimm.2004.02.014

[8] Riddell S, Greenberg P. T cell therapy of human CMV and EBV infection in immunocompromised hosts. Reviews in Medical Virology. 1997;7(3):181–92. 10398482 10.1002/(sici)1099-1654(199709)7:3<181::aid-rmv200>3.0.co;2-w

[9] Reusser P, Riddell SR, Meyers JD, Greenberg PD. Cytotoxic T-Lymphocyte response to cytomegalovirus after human allogeneic bone marrow transplantation: pattern of recovery and correlation with cytomegalovirus Infection and Disease. Blood. 1991;78(5):1371–80. 1652311

[10] Chen SF, Tu W-W, Sharp MA, Tongson EC, He X-S, Greenberg HB, et al Antiviral CD8 T cells in the control of primary human cytomegalovirus infection in early childhood. The Journal of Infectious Diseases. 2004;189(9):1619–27. doi: 10.1086/383249 15116298 10.1086/383249

[11] Sylwester A, Mitchell B, Edgar J, Taormina C, Pelte C, Ruchti F, et al Broadly targeted human cytomegalovirus-specific CD4+ and CD8+ T cells dominate the memory compartments of exposed subjects. The Journal of Experimental Medicine. 2005;202(5):673–85. doi: 10.1084/jem.20050882 16147978 10.1084/jem.20050882 PMC2212883

[12] Huygens A, Dauby N, Vermijlen D, Marchant A. Immunity to cytomegalovirus in early life. Frontiers in Immunology. 2014;5. 10.3389/fimmu.2014.00552 PMC4214201 25400639

[13] Kondo E, Akatsuka Y, Kuzushima K, Tsujimura K, Asakura S, Tajima K, et al Identification of novel CTL epitopes of CMV-pp65 presented by a variety of HLA alleles. Blood. 2004;103(2):630–8. doi: 10.1182/blood-2003-03-0824 12947002 10.1182/blood-2003-03-0824

[14] Wills MR, Okecha G, Weekes MP, Gandhi MK, Sissons PJG, Carmichael AJ. Identification of naive or antigen-experienced human CD8(+) T cells by expression of costimulation and chemokine receptors: analysis of the human cytomegalovirus-specific CD8(+) T cell response. The Journal of Immunology. 2002;168(11):5455–64. 12023339 10.4049/jimmunol.168.11.5455

[15] Akiyama Y, Maruyama K, Mochizuki T, Sasaki K, Takaue Y, Yamaguchi K. Identification of HLA-A24-restricted CTL epitope encoded by the matrix protein pp65 of human cytomegalovirus. Immunology Letters. 2002;83(1):21–30. 12057851 10.1016/s0165-2478(02)00073-1

[16] Khan N, Best D, Bruton R, Nayak L, Rickinson AB, Moss PA. T cell recognition patterns of immunodominant cytomegalovirus antigens in primary and persistent infection. The Journal of Immunology. 2007;178(7):4455–65. 17372003 10.4049/jimmunol.178.7.4455

[17] Kern F, Surel IP, Faulhaber N, Frömmel C, Schneider-Mergener J, Schönemann C, et al Target structures of the CD8(+)-T-cell response to human cytomegalovirus: the 72-kilodalton major immediate-early protein revisited. Journal of Virology. 1999;73(10):8179–84. 10482568 10.1128/jvi.73.10.8179-8184.1999 PMC112835

[18] Braendstrup P, Mortensen BK, Justesen S, Osterby T, Rasmussen M, Hansen AM, et al Identification and HLA-tetramer-validation of human CD4+ and CD8+ T cell responses against HCMV proteins IE1 and IE2. PLoS One. 2014;9(4):e94892 doi: 10.1371/journal.pone.0094892 24760079 10.1371/journal.pone.0094892 PMC3997423

[19] Crough T, Khanna R. Immunobiology of human cytomegalovirus: from bench to bedside. Clinical Microbiology Reviews. 2009;22(1):76–98. doi: 10.1128/CMR.00034-08 19136435 10.1128/CMR.00034-08 PMC2620639

[20] Adland E, Paioni P, Thobakgale C, Laker L, Mori L, Muenchhoff M, et al Discordant Impact of HLA on Viral Replicative Capacity and Disease Progression in Pediatric and Adult HIV Infection. PLoS Pathogens. 2015;11(6). 10.1371/journal.ppat.1004954 PMC4468173 26076345

[21] Payne R, Muenchhoff M, Mann J, Roberts HE, Matthews P, Adland E, et al Impact of HLA-driven HIV adaptation on virulence in populations of high HIV seroprevalence. PNAS. 2014;111(50):E5393–400. doi: 10.1073/pnas.1413339111 25453107 10.1073/pnas.1413339111 PMC4273423

[22] Payne RP, Kløverpris H, Sacha JB, Brumme Z, Brumme C, Buus S, et al Efficacious early antiviral activity of HIV Gag- and Pol-specific HLA-B*2705-restricted CD8+ T cells. Journal of Virology. 2010;84(20):10543–57. doi: 10.1128/JVI.00793-10 20686036 10.1128/JVI.00793-10 PMC2950555

[23] Cano P, Klitz W, Mack SJ, Maiers M, Marsh SGE, Noreen H, et al Common and well-documented HLA Alleles. Human Immunology. 2007;68(5):392–417. doi: 10.1016/j.humimm.2007.01.014 17462507 10.1016/j.humimm.2007.01.014

[24] Leisner C, Loeth N, Lamberth K, Justesen S, Sylvester-Hvid C, Schmidt EG, et al One-pot, mix-and-read peptide-MHC tetramers. PLoS ONE. 2008;3(2):e1678 doi: 10.1371/journal.pone.0001678 18301755 10.1371/journal.pone.0001678 PMC2244712

[25] Larsen EM, Kloverpris H, Stryhn A, Koofhethile CK, Sims S, Ndung’U T, et al HLArestrictor-a tool for patient-specific predictions of HLA restriction elements and optimal epitopes within peptides. Immunogenetics. 2011;63:43–55. doi: 10.1007/s00251-010-0493-5 21079948 10.1007/s00251-010-0493-5

[26] Weekes MP, Wills MR, Mynard KIM, Carmichael AJ, Sissons JGP. The memory cytotoxic T-lymphocyte (CTL) response to human cytomegalovirus infection contains individual peptide-specific CTL clones that have undergone extensive expansion in vivo. Journal of Virology. 1999;73(3):2099–108. 9971792 10.1128/jvi.73.3.2099-2108.1999 PMC104454

[27] Ameres S, Mautner J, Schlott F, Neuenhahn M, Busch DH, Plachter B, et al Presentation of an immunodominant Immediate-Early CD8+ T Cell epitope resists Human Cytomegalovirus immunoevasion. PLoS Pathogens. 2013;9(5). 10.1371/journal.ppat.1003383 PMC3662661 23717207

[28] Cao K, Hollenbach J, Shi X, Shi W, Chopek M, Fernández-Viña MA. Analysis of the frequencies of HLA-A, B, and C alleles and haplotypes in the five major ethnic groups of the United States reveals high levels of diversity in these loci and contrasting distribution patterns in these populations. Human Immunology. 2001;62(9):1009–30. 11543903 10.1016/s0198-8859(01)00298-1

[29] Gavin MA, Gilbert MJ, Riddell SR, Greenberg PD, Bevan MJ. Alkali hydrolysis of recombinant proteins allows for the rapid identification of class I MHC-restricted CTL epitopes. The Journal of immunology. 1993;151(8):3971–80. 7691936

[30] Rist M, Smith C, Bell MJ, Burrows SR, Khanna R. Cross-recognition of HLA DR4 alloantigen by virus-specific CD8+ T cells: a new paradigm for self-/ nonself-recognition. Blood. 2009;114(11):2244–53. doi: 10.1182/blood-2009-05-222596 19617574 10.1182/blood-2009-05-222596

[31] Longmate J, York J, Rosa CL, Krishnan R, Zhang M, Senitzer D, et al Population coverage by HLA class-I restricted cytotoxic T-lymphocyte epitopes. Immunogenetics. 2001;52(3–4):165–73. 11220618 10.1007/s002510000271

[32] Elkington R, Walker S, Crough T, Menzies M, Tellam J, Bharadwaj M, et al Ex vivo profiling of CD8+-T-cell responses to human cytomegalovirus reveals broad and multispecific reactivities in healthy virus carriers. Journal of Virology. 2003;77(9):5226–40. doi: 10.1128/JVI.77.9.5226-5240.2003 12692225 10.1128/JVI.77.9.5226-5240.2003 PMC153951

[33] Leslie A, Price DA, Mkhize P, Bishop K, Rathod A, Day C, et al Differential selection pressure exerted on HIV by CTL targeting identical epitopes but restricted by distinct HLA alleles from the same HLA supertype. The Journal of Immunology. 2006;177(7):4699–708. 16982909 10.4049/jimmunol.177.7.4699

[34] Carlson JM, Listgarten J, Pfeifer N, Tan V, Kadie C, Walker BD, et al Widespread impact of HLA restriction on immune control and escape pathways of HIV-1. Journal of Virology. 2012;86(9):5230–43. doi: 10.1128/JVI.06728-11 22379086 10.1128/JVI.06728-11 PMC3347390

[35] O’Hara GA, Welten SPM, Klenerman P, Arens R. Memory T cell inflation: Understanding cause and effect. Trends in Immunology. 2012;33(2):84–90. doi: 10.1016/j.it.2011.11.005 22222196 10.1016/j.it.2011.11.005

[36] Karrer U, Sierro S, Wagner M, Oxenius A, Hengel H, Koszinowski UH, et al Memory inflation: continuous accumulation of antiviral CD8+ T cells over time. Journal of Immunology. 2003;170(4):2022–9. 10.4049/jimmunol.170.4.2022 12574372

[37] Klenerman P, Oxenius A. T cell responses to cytomegalovirus. Nature Reviews Immunology. 2016;16(6):367–77. doi: 10.1038/nri.2016.38 27108521 10.1038/nri.2016.38

[38] Macdonald WA, Purcell AW, Mifsud NA, Ely LK, Williams DS, Chang L, et al A naturally selected dimorphism within the HLA-B44 supertype alters Class I structure, peptide repertoire, and T cell recognition. The Journal of Experimental Medicine. 2003;198(5):679–91. doi: 10.1084/jem.20030066 12939341 10.1084/jem.20030066 PMC2194191

[39] Kovacs A, Schluchter M, Easley K, Demmler G, Shearer W, Russa PL, et al Cytomegalovirus Infection and HIV-1 Disease Progression in Infants Born To HIV-1 –Infected Women Cytomegalovirus Infection and HIV-1 Disease Progression. The New England Journal of Medicine. 1999;341(2):77–84. 10395631 10.1056/NEJM199907083410203 PMC4280563

