# Supplementary figures and images for: Immunodominant cytomegalovirus-specific CD8+ T-cell responses in sub-Saharan African populations
Source: PLoS One. 2017 Dec 12;12(12):e0189612. doi: 10.1371/journal.pone.0189612 (PMC5726643; doi:10.1371/journal.pone.0189612)

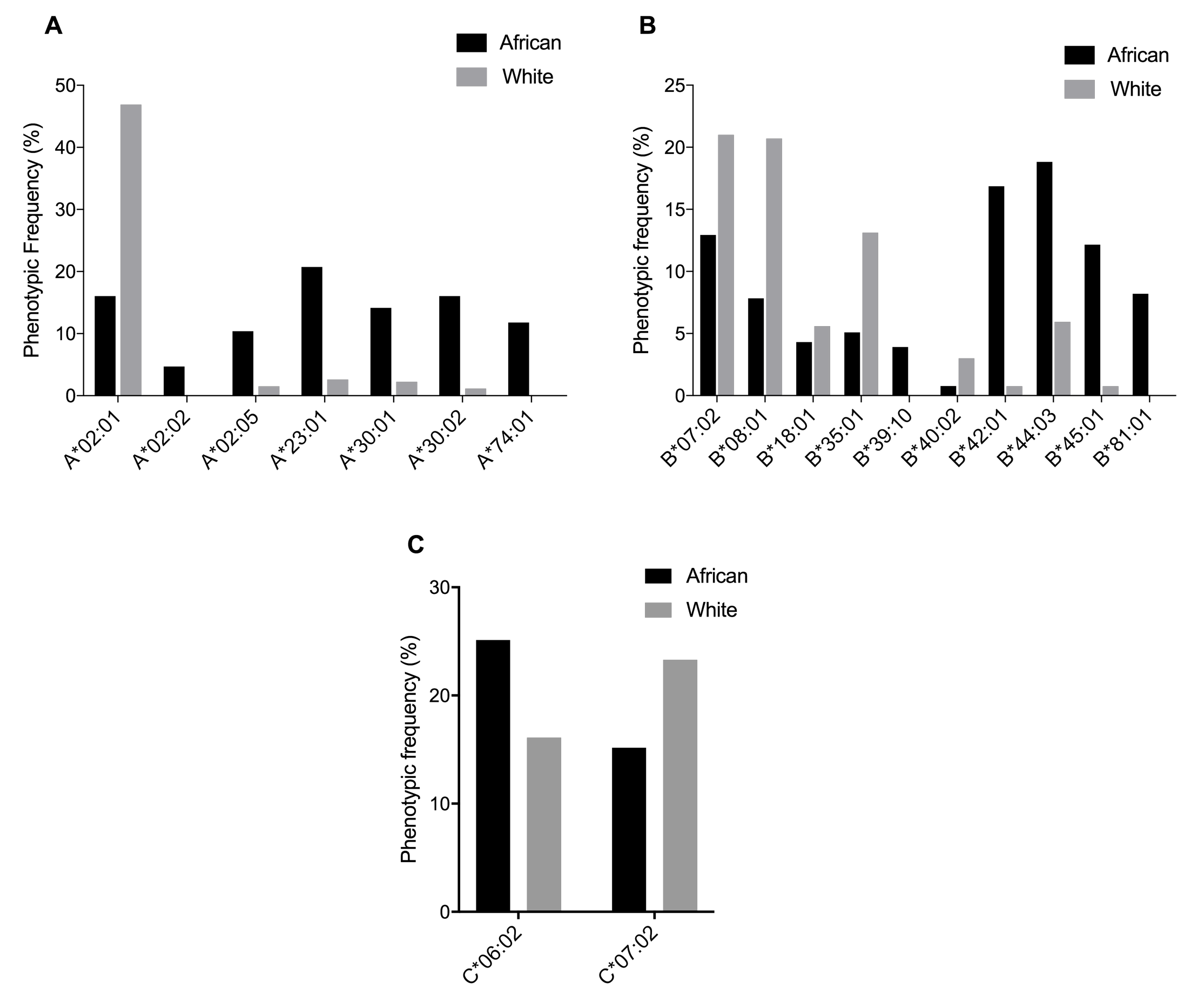

Supplement: S1 Fig — The figure showing the phenotypic frequencies of HLA alleles highlighted in Tables 2 and 3. (A) Phenotypic frequencies of HLA-A molecules, (B) phenotypic frequencies of HLA-B molecules and (C) phenotypic frequencies of HLA-C molecules in our African cohort and a representative white population [28]. (TIFF) [file pone.0189612.s001.tiff]

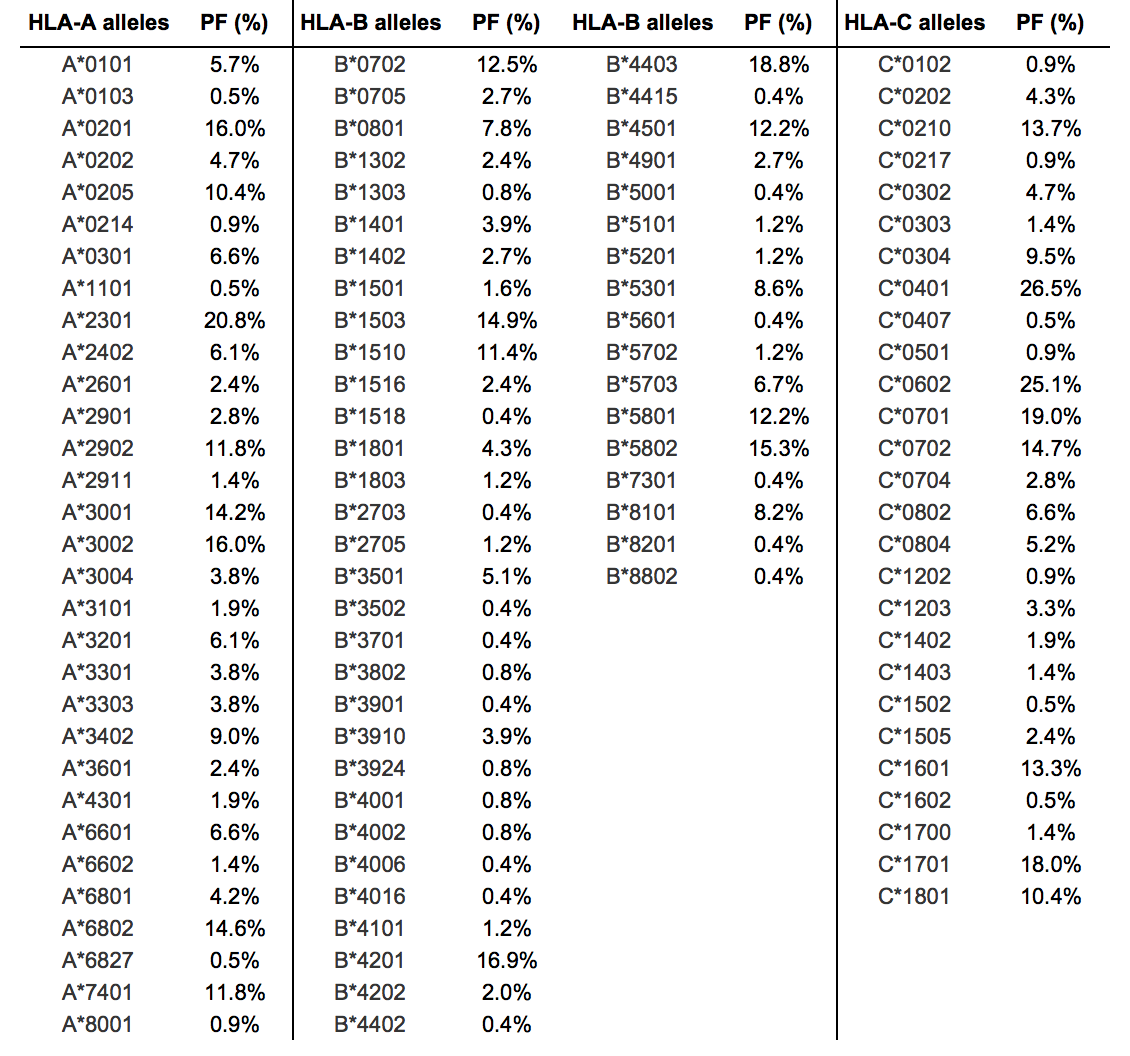

Supplement: S1 Table — (TIFF) [file pone.0189612.s002.tiff]
