# Supplementary material for: Immunodominant cytomegalovirus-specific CD8+ T-cell responses in sub-Saharan African populations
Source: PLoS One. 2017 Dec 12;12(12):e0189612. doi: 10.1371/journal.pone.0189612 (PMC5726643; doi:10.1371/journal.pone.0189612)
Supplement: S2 Table — (A) Sequences of overlapping peptides in pp65, (B) sequences of overlapping peptides in IE-1 and (C), sequences of overlapping peptides in IE-2. (PDF) [file pone.0189612.s003.pdf]

A) PP65

Peptide No.	Sequence
1	MESRGRRCPEMISVL
2	GRRCPEMISVLGPIS
3	PEMISVLGPISGHVL
4	SVLGPISGHVLKAVF
5	PISGHVLKAVFSRGD
6	HVLKAVFSRGDTPVL
7	AVFSRGDTPVLPHET
8	RGDTPVLPHETRLLQ
9	PVLPHETRLLQTGIOH
10	HETRLLQTGIHVRVS
11	LLQTGIHVRVSQPSL
12	GIHVRVSQPSLILVS
13	RVSQPSLILVSQYTP
14	PSLILVSQYTPDSTP
15	LVSQYTPDSTPCHRG
16	YTPDSTPCHRGDNQL
17	STPCHRGDNQLQVQOH
18	HRGDNQLQVQHTYFT
19	NQLQVQHTYFTGSEV
20	VQHTYFTGSEVENVS
21	YFTGSEVENVSVNVOH
22	SEVENVSVNVHNPTG
23	NVSVNVHNPTGRSIC
24	NVHNPTGRSICPSQE
25	PTGRSICPSQEPMSI
26	SICPSQEPMSIYVYA
27	SQEPMSIYVYALPLK
28	MSIYVYALPLKMLNI
29	VYALPLKMLNIPSIN
30	PLKMLNIPSINVHHY
31	LNIPSINVHHYPSAA
32	SINVHHYPSAAERKOH
33	HHYPSAAERKHRHLP
34	SAAERKHRHLPVADA
35	RKHRHLPVADAVIHA
36	HLPVADAVIHASGKQ
37	ADAVIHASGKQMWQA
38	IHASGKQMWQARLTV
39	GKQMWQARLTVSGLA
40	WQARLTVSGLAWTRQ
41	LTVSGLAWTRQQNQW
42	GLAWTRQQNQWKEPD
43	TRQQNQWKEPDVYYT
44	NQVVKEPDVYYTSAFV
45	EPDVYYTSAFVFPTK
46	YYTSAFVFPTKDVAL
47	AFVFPTKDVALRHW
48	PTKDVALRHWCAHE
49	VALRHWCAHELVCS
50	HWCAHELVCSMENT
51	AHELVCSMENTRATK
51	AHELVCSMENTRATK
52	VCSMENTRATKMQVI
53	ENTRATKMQVIGDQY
54	ATKMQVIGDQYVKVY
55	QVIGDQYVKVYLESF
56	DQYVKVYLESFCEDV
57	KVYLESFCEDVPSGK
58	ESFCEDVPSGKLFMOH
59	EDVPSGKLFMHVTLG
60	SGKLFMHVTLGSDVE
61	FMHVTLGSDVEEDLT
62	TLGSDVEEDLTMTRN
63	DVEEDLTMTRNPQPF
64	DLTMTRNPQPFMRPOH
65	TRNPQPFMRPHERNG
66	QPFMRPHERNGFTVL
67	RPHERNGFTVLCPKN
68	RNGFTVLCPKNMIIK
69	TVLCPKNMIIKPGKI
70	PKNMIIKPGKISHIM
71	IIKPGKISHIMLDVA
72	GKISHIMLDVAFTSOH
73	HIMLDVAFTSHEHFG
74	DVAFTSHEHFGLLCP
75	TSHEHFGLLCPKSIP
76	HFGLLCPKSIPGLSI
77	LCPKSIPGLSISGNL
78	SIPGLSISGNLLMNG
79	LSISGNLLMNGQQIF
80	GNLLMNGQQIFLEVQ
81	MNGQQIFLEVQAIRE
82	QIFLEVQAIRETVEL
83	EVQAIRETVELRQYD
84	IRETVELRQYDPVAA
85	VELRQYDPVAALFFF
86	QYDPVAALFFFDIDL
87	VAALFFFDIDLLLQR
88	FFFDIDLLLQRGPQY
89	IDLLLQRGPQYSEHP
90	LQRGPQYSEHPTFTS
91	PQYSEHPTFTSQYRI
92	EHPTFTSQYRIQGKL
93	FTSQYRIQGKLEYROH
94	YRIQGKLEYRHTWDR
95	GKLEYRHTWDRHDEG
96	YRHTWDRHDEGAAQG
97	WDRHDEGAAQGDDDV
98	DEGAAQGDDDVWTSG
99	AQGDDDVWTSGSDSD
100	DDVWTSGSDSDEELV
101	TSGSDSDEELVTTER
102	DSDEELVTTERKTPR
103	ELVTTERKTPRVTGG
104	TERKTPRVTGGGAMA
105	TPRVTGGGAMAGAST
106	TGGGAMAGASTSAGR
107	AMAGASTSAGRKRKS
108	ASTSAGRKRKSASSA
109	AGRKRKSASSATACT
110	RKSASSATACTSGVM
111	SSATACTSGVMTRGR
112	ACTSGVMTRGRLKAE
113	GVMTRGRLKAESTVA
114	RGRLKAESTVAPEED
115	KAESTVAPEEDTDED
116	TVAPEEDTDEDSDNE
117	EEDTDEDSDNEIHNP
118	DEDSDNEIHNPAVFT
119	DNEIHNPAVFTWPPW
120	HNPAVFTWPPWQAGI
121	VFTWPPWQAGILARN
122	PPWQAGILARNLVPM
123	AGILARNLVPMVATV
124	ARNLVPMVATVQGQN
125	VPMVATVQGQNLKYQ
126	ATVQGQNLKYQEFFW
127	GQNLKYQEFFWDAND
128	KYQEFFWDANDIYRI
129	FFWDANDIYRIFAEL
130	ANDIYRIFAELEGVW
131	YRIFAELEGVWQPAA
132	AELEGVWQPAAQPKR
133	GVWQPAAQPKRRRHR
134	PAAQPKRRRHRQOAL
135	PKRRRHRQOALPGPC
136	RHRQOALPGPCIAST
137	OALPGPCIASTPKKOH
138	LPGPCIASTPKKHRG

B) IE1

Peptide No.	Sequence
139	MESSAKRKMDPDNPD
140	AKRKMDPDNPDEGPS
141	MDPDNPDEGPSSKVP
142	NPDEGPSSKVPRPET
143	GPSSKVPRPETPVTK
144	KVPRPETPVTKATTF
145	PETPVTKATTFLQTM
146	VTKATTFLQTMLRKE
147	TTFLQTMLRKEVNSQ
148	QTMLRKEVNSQLSLG
149	RKEVNSQLSLGDPLF
150	NSQLSLGDPLFPELA
151	SLGDPLFPELAEESL
152	PLFPELAEESLKTFE
153	ELAEESLKTFEQVTE
154	ESLKTFEQVTEDCNE
155	TFEQVTEDCNENPEK
156	VTEDCNENPEKDVLA
157	CNENPEKDVLAELVK
158	PEKDVLAELVKQIKV
159	VLAELVKQIKVRVDM
160	LVKQIKVRVDMVRHR
161	IKVRVDMVRHRIKEH
162	VDMVRHRIKEHMLKK
163	RHRIKEHMLKKYTQT
164	KEHMLKKYTQTEEKF
165	LKKYTQTEEKFTGAF
166	TQTEEKFTGAFNMMG
167	EKFTGAFNMMGGCLQ
168	GAFNMMGGCLQNALD
169	MMGGCLQNALDILDK
170	CLQNALDILDKVHEP
171	ALDILDKVHEPFEEM
172	LDKVHEPFEEMKCIG
173	HEPFEEMKCIGLTMQ
174	EEMKCIGLTMQSMYE
175	CIGLTMQSMYENYIV
176	TMQSMYENYIVPEDK
177	MYENYIVPEDKREMW
178	YIVPEDKREMWMACI
179	EDKREMWMACIKELH
180	EMWMACIKELHDVSK
181	ACIKELHDVSKGAAN
182	ELHDVSKGAANKLGG
183	VSKGAANKLGGALQA
184	AANKLGGALQAKARA
185	LGGALQAKARAKKDE
186	LQAKARAKKDELRRK
187	ARAKKDELRRKMMYM
188	KDELRRKMMYMCYRN
189	RRKMMYMCYRNIEFF
190	MYMCYRNIEFFTKNS
191	YRNIEFFTKNSAFPK
192	EFFTKNSAFPKTTNG
193	KNSAFPKTTNGCSQA
194	FPKTTNGCSQAMAAL
195	TNGCSQAMAALQNLP
196	SQAMAALQNLPQCSP
197	AALQNLPQCSPDEIM
198	NLPQCSPDEIMAYAQ
199	CSPDEIMAYAQKIFK
200	EIMAYAQKIFKILDE
201	YAQKIFKILDEERDK
202	IFKILDEERDKVLTH
203	LDEERDKVLTHIDHI
204	RDKVLTHIDHIFMDI
205	LTHIDHIFMDILTTC
206	DHIFMDILTTCVETM
207	MDILTTCVETMCNEY
208	TTCVETMCNEYKVTS
209	ETMCNEYKVTSDACM
210	NEYKVTSDACMMTMY
211	VTSDACMMTMYGGIS
212	ACMMTMYGGISLLSE
213	TMYGGISLLSEFCRV
214	GISLLSEFCRVLCCY
215	LSEFCRVLCCYVLEE
216	CRVLCCYVLEETSVM
217	CCYVLEETSVMLAKR
218	LEETSVMLAKRPLIT
219	SVMLAKRPLITKPEV
220	AKRPLITKPEVISVM
221	LITKPEVISVMKRRI
222	PEVISVMKRRIEEIC
223	SVMKRRIEEICMKVF
224	RRIEEICMKVFAQYI
225	EICMKVFAQYILGAD
226	KVFAQYILGADPLRV
227	QYILGADPLRVCSPS
228	GADPLRVCSPSVDDL
229	LRVCSPSVDDLRAIA
230	SPSVDDLRAIAEESD
231	DDLRAIAEESDEEEA
232	AIAEESDEEEAIVAY
233	ESDEEEAIVAYTLAT
234	EEAIVAYTLATAGVS
235	VAYTLATAGVSSSDS
236	LATAGVSSSDSLVSP
237	GVSSSDSLVSPPESP
238	SDSLVSPPESPVPAT
239	VSPPESPVPATIPLS
240	ESPVPATIPLSSVIV
241	PATIPLSSVIVAENS
242	PLSSVIVAENSDQEE
243	VIVAENSDQEESEQS
244	ENSDQEESEQSDEEE
245	QEESEQSDEEEEEGA
246	EQSDEEEEEGAQEER
247	EEEEEGAQEEREDTV
248	EGAQEEREDTVSVKS
249	EEREDTVSVKSEPVS
250	DTVSVKSEPVSEIEE
251	VKSEPVSEIEEVAPE
252	PVSEIEEVAPEEEED
253	IEEVAPEEEEDGAEE
254	APEEEEDGAEEPTAS
255	EEDGAEEPTASGGKS
256	AEEPTASGGKSTHPM
257	TASGGKSTHPMVTRS
258	GKSTHPMVTRSKADQ

C) IE2

Peptide No.	Sequence
259	MESSAKRKMDPDNPD
260	AKRKMDPDNPDEGPS
261	MDPDNPDEGPSSKVP
262	NPDEGPSSKVPRPET
263	GPSSKVPRPETPVTK
264	KVPRPETPVTKATTF
265	PETPVTKATTFLQTM
266	VTKATTFLQTMLRKE
267	TTFLQTMLRKEVNSQ
268	QTMLRKEVNSQLSLG
269	RKEVNSQLSLGDPLF
270	NSQLSLGDPLFPELA
271	SLGDPLFPELAEESL
272	PLFPELAEESLKTFE
273	ELAEESLKTFEQVTE
274	ESLKTFEQVTEDCNE
275	TFEQVTEDCNENPEK
276	VTEDCNENPEKDVLA
277	CNENPEKDVLAELGD
278	PEKDVLAELGDILAQ
279	VLAELGDILAQAVNH
280	LGDILAQAVNHAGID
281	LAQAVNHAGIDSSST
282	VNHAGIDSSSTGPTL
283	GIDSSSTGPTLTTHS
284	SSTGPTLTTHSCSVS
285	PTLTTHSCSVSSAPL
286	THSCSVSSAPLNKPT
287	SVSSAPLNKPTPTSV
288	APLNKPTPTSVAVTN
289	KPTPTSVAVTNTPLP
290	TSVAVTNTPLPGASA
291	VTNTPLPGASATPEL
292	PLPGASATPELSPRK
293	ASATPELSPRKKPRK
294	PELSPRKKPRKTTRP
295	PRKKPRKTTRPFKVI
296	PRKTTRPFKVIIKPP
297	TRPFKVIIKPPVPPA
298	KVIIKPPVPPAPIML
299	KPPVPPAPIMLPLIK
300	PPAPIMLPLIKQEDI
301	IMLPLIKQEDIKPEP
302	LIKQEDIKPEPDFTI
303	EDIKPEPDFTIQYRN
304	PEPDFTIQYRNKIID
305	FTIQYRNKIIDTAGC
306	YRNKIIDTAGCIVIS
307	IIDTAGCIVISDSEE
308	AGCIVISDSEEEQGE
309	VISDSEEEQGEEVET
310	SEEEQGEEVETRGAT
311	QGEEVETRGATASSP
312	VETRGATASSPSTGS
313	GATASSPSTGSGTPR
314	SSPSTGSGTPRVTSP
315	TGSGTPRVTSPTHPL
316	TPRVTSPTHPLSQMN
317	TSPTHPLSQMNHPPL
318	HPLSQMNHPPLPDPL
319	QMNHPPLPDPLGRPD
320	PPLPDPLGRPDEDSS
321	DPLGRPDEDSSSSSS
322	RPDEDSSSSSSSSCS
323	DSSSSSSSSCSSASD
324	SSSSSCSSASDSESE
325	SCSSASDSESESEEM
326	ASDSESESEEMKCSS
327	ESESEEMKCSSGGGA
328	EEMKCSSGGGASVTS
329	CSSGGGASVTSSHHG
330	GGASVTSSHHGRGGF
331	VTSSHHGRGGFGGAA
332	HHGRGGFGGAASSSL
333	GGFGGAASSSLLSCG
334	GAASSSLLSCGHQSS
335	SSLLSCGHQSSGGAS
336	SCGHQSSGGASTGPR
337	QSSGGASTGPRKKKS
338	GASTGPRKKKSKRIS
339	GPRKKKSKRISELDN
340	KKSKRISELDNEKVR
341	RISELDNEKVRNIMK
342	LDNEKVRNIMKDKNT
343	KVRNIMKDKNTPFCT
344	IMKDKNTPFCTPNVQ
345	KNTPFCTPNVQTRRG
346	FCTPNVQTRRGRVKI
347	NVQTRRGRVKIDEVS
348	RRGRVKIDEVSRMFR
349	VKIDEVSRMFRNTNR
350	EVSRMFRNTNRSLEY
351	MFRNTNRSLEYKNLP
352	TNRSLEYKNLPFTIP
353	LEYKNLPFTIPSMHQ
354	NLPFTIPSMHQVLDE
355	TIPSMHQVLDEAIKA
356	MHQVLDEAIKACKTM
357	LDEAIKACKTMQVNN
358	IKACKTMQVNNKGIQ
359	KTMQVNNKGIQIIYT
360	VNNKGIQIIYTRNHE
361	GIQIIYTRNHEVKSE
362	IYTRNHEVKSEVDAV
363	NHEVKSEVDAVRCRL
364	KSEVDAVRCRLGTMC
365	DAVRCRLGTMCNLAL
366	CRLGTMCNLALSTPF
367	TMCNLALSTPFLMEH
368	LALSTPFLMEHTMPV
369	TPFLMEHTMPVTHPP
370	MEHTMPVTHPPEVAQ
371	MPVTHPPEVAQRTAD
372	HPPEVAQRTADACNE
373	VAQRTADACNEGVKA
374	TADACNEGVKAAWSL
375	CNEGVKAAWSLKELH
376	VKAAWSLKELHTHQL
377	WSLKELHTHQLCPRS
378	ELHTHQLCPRSSDYR
379	HQLCPRSSDYRNMII
380	PRSSDYRNMIIHAAT
381	DYRNMIIHAATPVDL
382	MIIHAATPVDLLGAL
383	AATPVDLLGALNLCL
384	VDLLGALNLCLPLMQ
385	GALNLCLPLMQKFPK
386	LCLPLMQKFPKQVMV
387	LMQKFPKQVMVRIFS
388	FPKQVMVRIFSTNQG
389	VMVRIFSTNQGGFML
390	IFSTNQGGFMLPIYE
391	NQGGFMLPIYETAAK
392	FMLPIYETAAKAYAV
393	IYETAAKAYAVGQFE
394	AAKAYAVGQFEQPTE
395	YAVGQFEQPTETPPE
396	QFEQPTETPPEDLDT
397	PTETPPEDLDTLSLA
398	PPEDLDTLSLAIEAA
399	LDTLSLAIEAAIQDL
400	SLAIEAAIQDLRNKS
401	LAIEAAIQDLRNKSQ
